# Supplementary material for: Cancer-Related Mutations Are Not Enriched in Naive Human Pluripotent Stem Cells
Source: Cell Stem Cell. 2021 Jan 7;28(1):164–169.e2. doi: 10.1016/j.stem.2020.11.014 (PMC7837212; doi:10.1016/j.stem.2020.11.014)
Supplement: Document S1. Figures S1 and S2 and Tables S1 and S2 [file mmc1.pdf]

Cell Stem Cell, Volume *28*

Supplemental Information

Cancer-Related Mutations Are Not Enriched in Naive Human Pluripotent Stem Cells

Giuliano Giuseppe Stirparo, Austin Smith, and Ge Guo

SUPPLEMENTAL LEGENDS

Supplement Figure 1

(A) Schematic of the pipeline used for the identification of SNPs from RNA-seq data.Variants were intersected with 43 cancer-related SNPs from Avior (2019) before and after application of hard-filtering.

(B) Total number of cancer-associated SNPs from Avior (2019) identified in HNES1 naïve cells on MEF or laminin substrates.

(C) Overlap between cancer associated SNPs from Avior et al (2019) identified in cR-S6EOS and HNES1 cells on MEF.

(D) Scatter plots of mapped reads in Avior et al. (2019) and this study.

(E) Schematic of the pipeline used for the identification of SNPs in RNA-seq data with and without removal of mouse reads by XenofilteR.

(F) Distribution of percentage of mouse reads for all naïve and primed hPSC samples.

Supplement Figure 2

(A) Heatmap with log2 expression value for cancer-associated genes in hPSCs before (CTRL) and after removal of mouse reads (XEN).

(B) PCA plots computed for all samples with all expressed protein coding genes. Left panel, before removal of mouse reads, right panel, after XenofilteR. N, naïve; P, primed as assigned by Avior et al. (2019). Purported naïve samples from Chan, Lee and Sperber align with conventional primed cells, as noted in previous analyses (Bredenkamp et al., 2019b; Takashima et al., 2014)

(C) Total number of cancer-associated SNPs detected in naïve hPSCs with different analyses.

Supplement Table 1

Summary of SNP analysis, showing datasets and samples analysed in this study and in Avior (2019). na denotes not analysed by Avior (2019). Note: cancer-related SNPs denote the 43 SNPs reported in Avior (2019)

Supplement Table 2

Table showing sample distribution of the 43 cancer-associated SNPs identified in Avior (2019) as determined in this study. The list of SNPs was downloaded from Supplementary Table 2 in Avior (2019) and include SNPs identified in hESC, iPSC or mesenchymal stromal cell (MSC) samples.

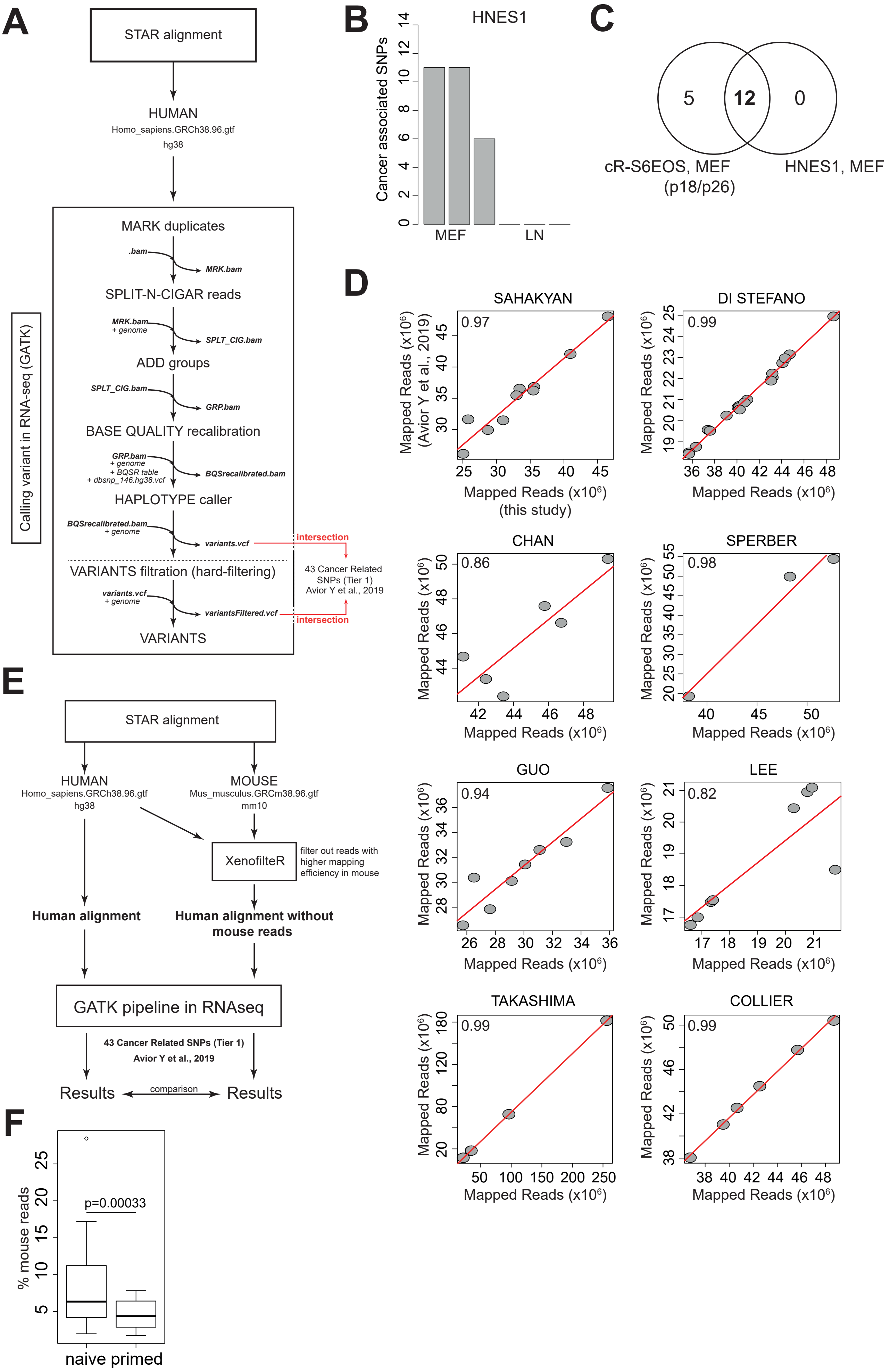
A
STAR alignment
HUMAN
Homo_sapiens.GRCh38.96.gtf
hg38
Calling variant in RNA-seq (GATK)
MARK duplicates
.bam
MRK.bam
SPLIT-N-CIGAR reads
MRK.bam
+ genome
SPLT_CIG.bam
ADD groups
SPLT_CIG.bam
GRP.bam
BASE QUALITY recalibration
GRP.bam
+ genome
+ BQSR table
+ dbsnp_146.hg38.vcf
BQSrecalibrated.bam
HAPLOTYPE caller
BQSrecalibrated.bam
+ genome
variants.vcf
intersection
43 Cancer Related SNPs (Tier 1) Avior Y et al., 2019
VARIANTS filtration (hard-filtering)
variants.vcf
+ genome
variantsFiltered.vcf
intersection
VARIANTS
B
HNES1
Cancer associated SNPs
MEF
LN
C
5
12
0
cR-S6EOS, MEF (p18/p26)
HNES1, MEF
D
SAHAKYAN
0.97
Mapped Reads (x10⁶) (Avior Y et al., 2019)
Mapped Reads (x10⁶) (this study)
DI STEFANO
0.99
CHAN
0.86
SPERBER
0.98
GUO
0.94
LEE
0.82
TAKASHIMA
0.99
COLLIER
0.99
Mapped Reads (x10⁶)
E
STAR alignment
HUMAN
Homo_sapiens.GRCh38.96.gtf
hg38
MOUSE
Mus_musculus.GRCm38.96.gtf
mm10
XenofilteR
filter out reads with higher mapping efficiency in mouse
Human alignment
Human alignment without mouse reads
GATK pipeline in RNAseq
43 Cancer Related SNPs (Tier 1)
Avior Y et al., 2019
Results
comparison
Results
F
% mouse reads
p=0.00033
naive primed

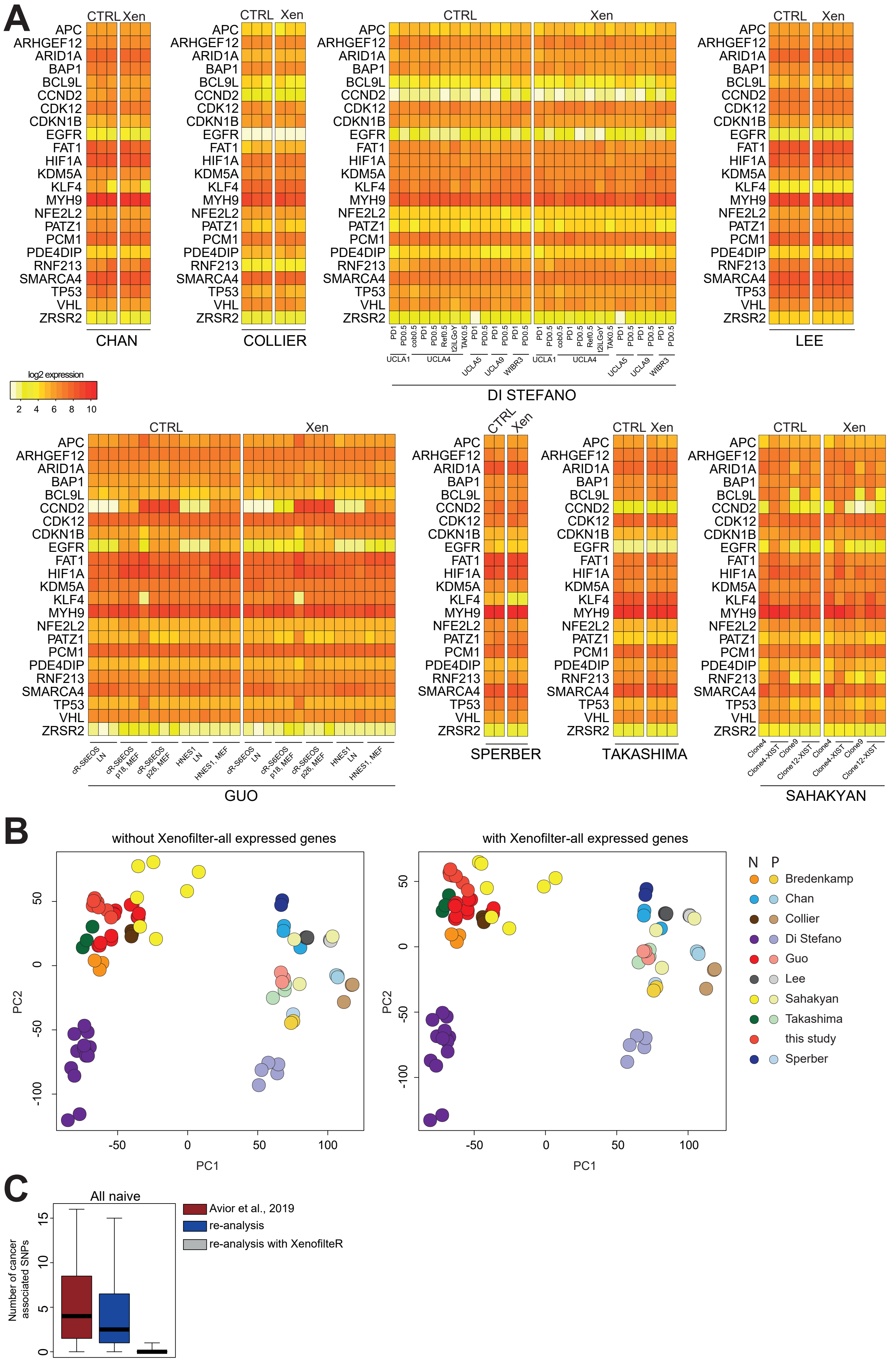
A
CTRL
Xen
APC
ARHGEF12
ARID1A
BAP1
BCL9L
CCND2
CDK12
CDKN1B
EGFR
FAT1
HIF1A
KDM5A
KLF4
MYH9
NFE2L2
PATZ1
PCM1
PDE4DIP
RNF213
SMARCA4
TP53
VHL
ZRSR2
CHAN
COLLIER
DI STEFANO
UCLA1
UCLA4
UCLA5
UCLA9
WIBR3
LEE
log2 expression
2 4 6 8 10
GUO
cR-S6EOS LN
cR-S6EOS p18, MEF
cR-S6EOS p26, MEF
HNES1 LN
HNES1, MEF
SPERBER
TAKASHIMA
SAHAKYAN
Clone4
Clone4-XIST
Clone9
Clone12-XIST
B
without Xenofilter-all expressed genes
with Xenofilter-all expressed genes
PC1
PC2
N P
Bredenkamp
Chan
Collier
Di Stefano
Guo
Lee
Sahakyan
Takashima
this study
Sperber
C
All naive
Number of cancer associated SNPs
Avior et al., 2019
re-analysis
re-analysis with XenofilteR
