# Supplementary material for: Cancer-Related Mutations Are Not Enriched in Naive Human Pluripotent Stem Cells
Source: Cell Stem Cell. 2021 Jan 7;28(1):164–169.e2. doi: 10.1016/j.stem.2020.11.014 (PMC7837212; doi:10.1016/j.stem.2020.11.014)
Supplement: Document S2. Article plus Supplemental Information [file mmc4.pdf]

Cell Stem Cell

Matters Arising

Cancer-Related Mutations Are Not Enriched in Naive Human Pluripotent Stem Cells

Highlights

- Culture conditions for naive stem cells do not select for cancer-related mutations
- Contaminating sequences in RNA-seq data can be misidentified as point mutations
- Filtering to remove mouse sequences eliminates most mutations
- Remaining mutations in naive stem cells are carried over from primed stem cells

Authors

Giuliano Giuseppe Stirparo, Austin Smith, Ge Guo

Correspondence

austin.smith@exeter.ac.uk (A.S.),
g.guo@exeter.ac.uk (G.G.)

In Brief

Acquisition of pathogenic mutations during in vitro propagation is a concern for applications of pluripotent stem cells. In this paper, Stirparo and colleagues show that detection of multiple mutations in naïve stem cells by RNA-seq analysis is due to contamination with mouse sequences. The actual cancer mutation incidence is low.

Stirparo et al., 2021, Cell Stem Cell *28*, 164–169
January 7, 2021 © 2020 The Authors. Published by Elsevier Inc.
https://doi.org/10.1016/j.stem.2020.11.014

CellPress

CellPress
OPEN ACCESS

Cell Stem Cell

Matters Arising

Cancer-Related Mutations Are Not Enriched in Naive Human Pluripotent Stem Cells

Giuliano Giuseppe Stirparo,[1,2] Austin Smith,[1,2,3,*] and Ge Guo[1,2,4,*]
[1]Wellcome-MRC Cambridge Stem Cell Institute, Jeffrey Cheah Biomedical Centre, University of Cambridge, Cambridge, CB2 0AW, UK
[2]Living Systems Institute, University of Exeter, Exeter, EX4 4QD, UK
[3]Department of Biochemistry, University of Cambridge, Cambridge, CB2 1QR, UK
[4]Lead Contact
*Correspondence: austin.smith@exeter.ac.uk (A.S.), g.guo@exeter.ac.uk (G.G.)
https://doi.org/10.1016/j.stem.2020.11.014

SUMMARY

Previous analysis of RNA sequencing (RNA-seq) data from human naive pluripotent stem cells reported multiple point "mutations" in cancer-related genes and implicated selective culture conditions. We observed, however, that those mutations were only present in co-cultures with mouse feeder cells. Inspection of reads containing the polymorphisms revealed complete identity to the mouse reference genome. After we filtered reads to remove sequences of mouse origin, the actual incidence of oncogenic polymorphisms arising in naive pluripotent stem cells is close to zero.

INTRODUCTION

An important consideration for the use of human pluripotent stem cells (hPSCs) in biomedical research and regenerative medicine is the acquisition of mutations, in particular in genes associated with cancer. This issue was highlighted in a recent study that reported point mutations in many cancer-related genes in one-third of hPSC lines (Avior et al., 2019). Using RNA sequencing (RNA-seq) data from a large panel of primed and naive hPSCs, Avior et al. (2019) discovered recurrent non-synonymous single nucleotide polymorphisms (SNPs) in multiple tier 1 cancer genes. Of particular note, the authors highlighted a 4-fold higher incidence of these mutations in naive hPSCs than in primed hPSCs. Naive cells are maintained by chemical inhibition of several signaling pathways (Dong et al., 2019), and Avior et al. (2019) proposed that oncogenic mutations are selected for because they confer a growth advantage in the presence of the inhibitors. The finding of mutations in genes linked to growth and cancer raises potentially grave concerns about consequences for *in vitro* phenotypes and *in vivo* tumorigenicity.

The study by Avior et al. (2019) included analysis of some samples from a dataset deposited by our laboratory (Guo et al., 2017). They reported the detection of mutations in *TP53* and other genes in the naive cell line cR-S6EOS. In our initial characterization of cR-S6EOS, we did not observe the four functionally validated dominant-negative mutations in *TP53* that had previously been detected in a number of conventional hPSCs (Merkle et al., 2017). To clarify the prevalence of cancer-related mutations in naive hPSCs, we re-examined RNA-seq data from different cultures of cR-S6EOS and other naive cell lines.

RESULTS

We first inspected the existence of the cancer-related mutations reported by Avior et al. (2019) in our cR-S6EOS dataset (Guo et al., 2017). We applied the established GATK pipeline for calling SNPs from RNA-seq data (McKenna et al., 2010; Figure S1A) and detected an average of ~14,000 SNPs. However, the mutations reported by Avior et al. (2019) were not present (Table S1). We reasoned that failure to detect these point mutations may have been attributable to our use of the optional variants hard-filtering step, which was designed to increase the stringency of SNP calls. Indeed, when we omitted the hard-filtering step, we detected a similar number of cancer-related mutations as reported by Avior et al. (2019). We identified a total of 17 of the Avior SNPs across all the replicates of cR-S6EOS at 2 different passage numbers (Table S1). We therefore applied the pipeline without the hard-filtering step to analyze additional samples in our previously deposited dataset.

The data are from naive cells in two culture conditions: (1) maintained on feeder layers of mouse embryo fibroblasts (MEFs); and (2) transferred from MEFs onto laminin for more than three passages. Cultures were of similar total passage number, and libraries were prepared and sequenced in parallel (Guo et al., 2017). Remarkably, however, in cR-S6EOS cultures on laminin, we did not detect any of the cancer-related SNPs identified in the MEF co-cultures (Figure 1A). We examined coverage per base of three SNPs identified by Avior et al. (2019) in *TP53*, *FAT1*, and *SMARCA4*. The SNPs were present in a fraction of reads from MEF cultures but completely absent from laminin samples (Figure 1B). Strikingly, in addition to the non-synonymous SNPs highlighted by Avior et al. (2019), we noted multiple nearby SNPs in samples from cultures on MEFs that were likewise completely absent in the laminin cultures.

These observations are counter-intuitive, particularly as the transition to feeder-free culture would be expected to impose stress and increase selective pressure. Moreover, the collective presence or absence of multiple SNPs in multiple genes in the same cells is not consistent with natural selection. We repeated the analysis for the embryo-derived naive cell line HNES1 (Guo

 © 2020 The Authors. Published by Elsevier Inc.
This is an open access article under the CC BY license (http://creativecommons.org/licenses/by/4.0/).

CellPress
OPEN ACCESS

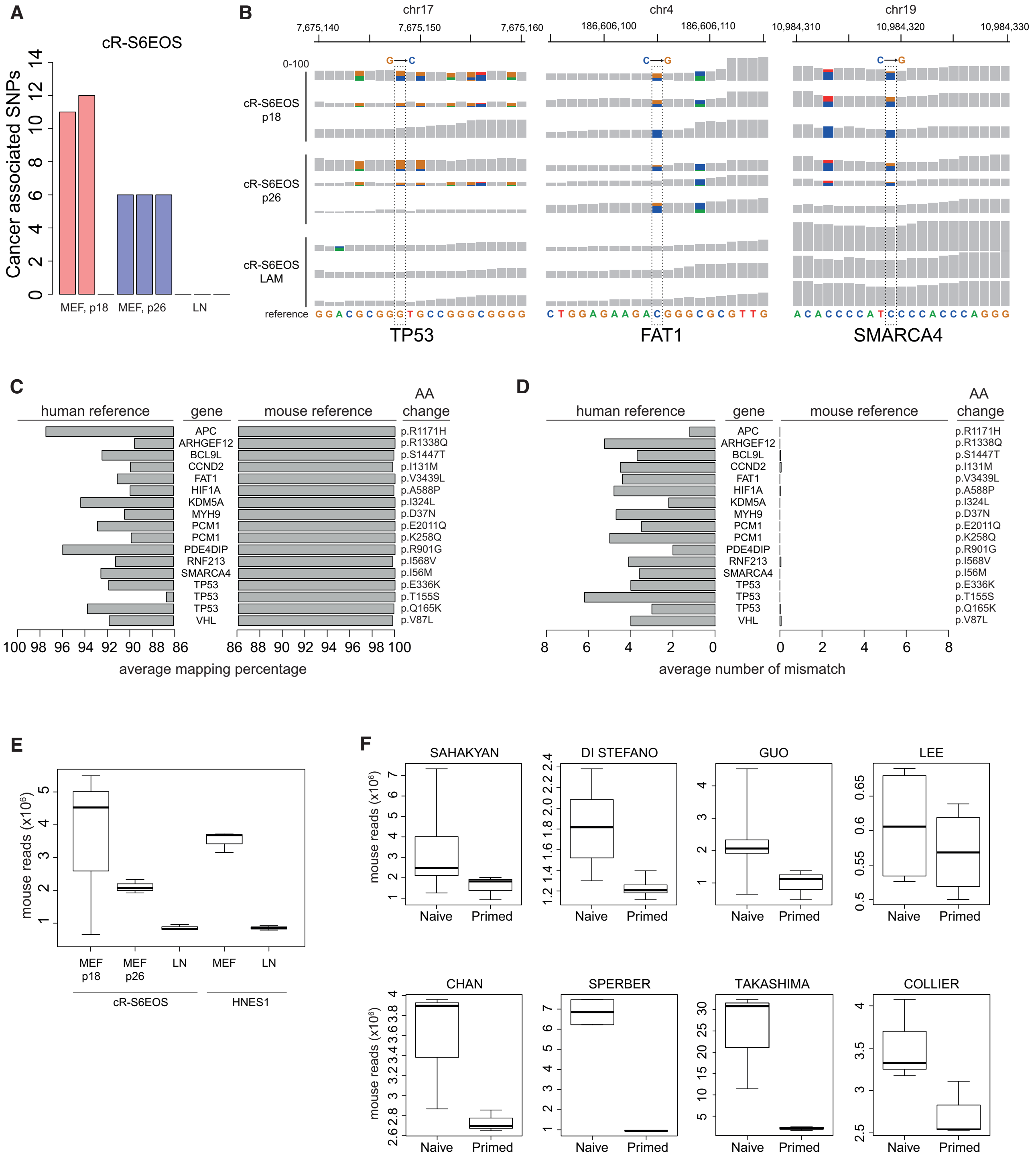

Figure 1. Analysis of Genomic Origin of SNPs Reported by Avior et al. (2019)

(A) Numbers of cancer-associated SNPs from Avior et al. (2019) in cR-S6EOS samples cultured on mouse embryo fibroblast (MEF) feeder layers or on laminin (LN) detected by the GATK pipeline without variants hard-filtering step.

(B) Integrative Genome Browser screenshot of selected cancer-associated SNPs from Avior et al. (2019) showing per base read coverage (0–100) in cR-S6EOS cultures on MEF or LN. Dotted lines highlight the SNP reported by Avior et al. (2019). Positions with alternative nucleotides are represented using different colors.

(C) Average mapping percentage of total reads from cR-S6EOS(MEF) samples harboring the indicated SNPs reported by Avior et al. (2019) when aligned against human or mouse reference sequences. See also Tables S1 and S2.

(D) Number of mismatches in reads as in (C) aligned against human or mouse reference sequences.

(E) Boxplots of the number of mouse reads detected by XenofilteR in naive cell samples from cultures on MEF or LN.

(F) Boxplots of the number of mouse reads identified by XenofilteR in naive and primed conditions across different datasets analyzed in Avior et al. (2019).

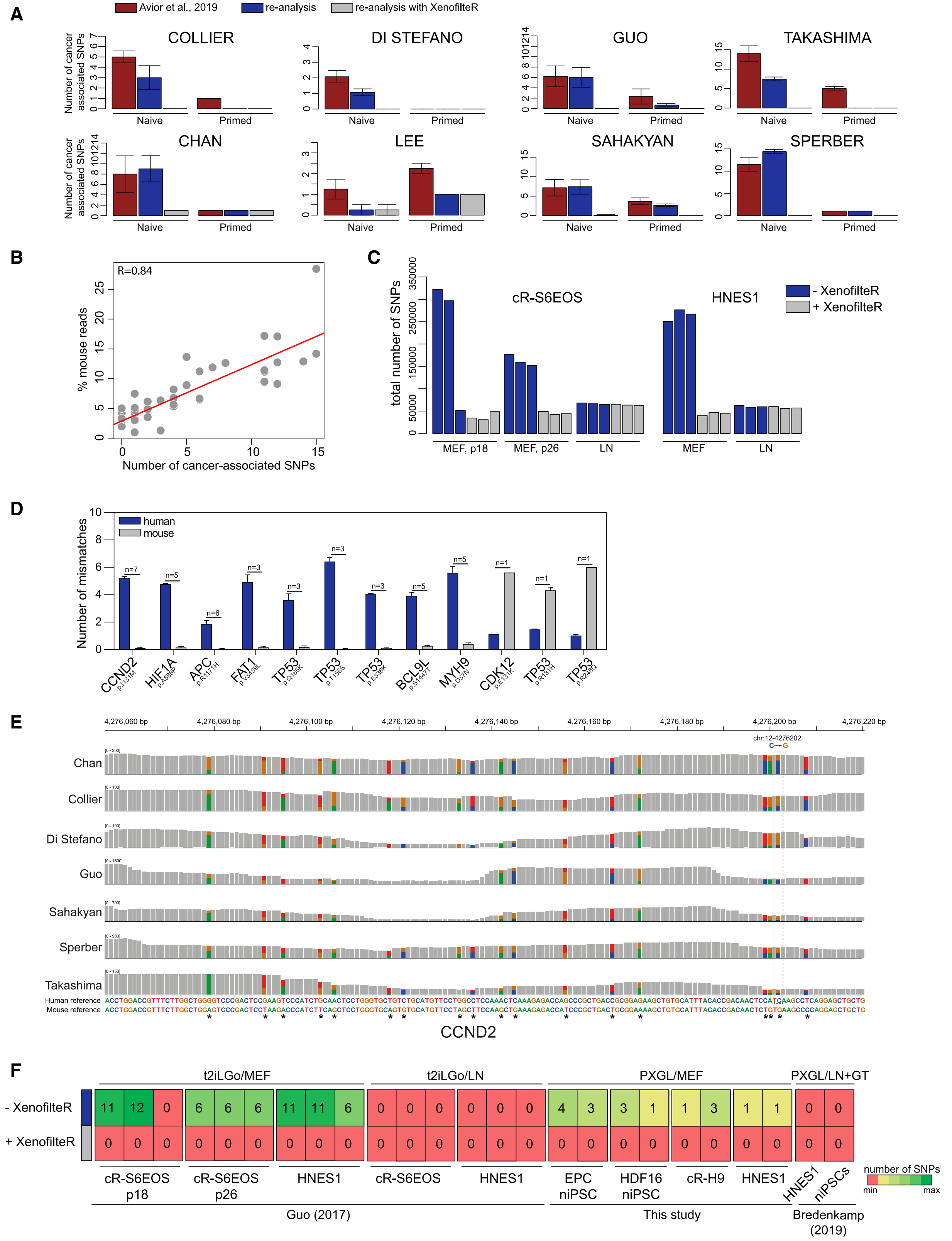

(legend on next page)

et al., 2016) and again found that the cancer-related mutations reported by Avior et al. (2019) were detected only in MEF cultures and not under feeder-free conditions (Figure S1B). We were further intrigued by a significant overlap in the cancer-related SNPs identified in MEF cultures between two entirely independent naive cell lines, namely, one generated by resetting and the other embryo derived (Guo et al., 2016, 2017; Figure S1C). Each of the Avior et al. (2019) SNPs identified in HNES1 is also present in cR-S6EOS. It is improbable that cell lines of independent genetic origins would show such a high number of identical mutations and that they would only be present in co-cultures with MEFs.

These observations prompted us to investigate whether contaminating MEF-derived sequences may contribute to SNP calls. We retrieved sequence reads harboring SNPs reported by Avior et al. (2019) that are detectable in cR-S6EOS MEF samples. These comprise 17 non-synonymous SNPs in 14 genes (Figure 1C). Alignment with the reference human and mouse gene sequences revealed that these reads have an average of >99% identity with mouse, higher than with human sequences. In all cases, the SNPs reported by Avior et al. (2019) match mouse gene sequences. Notably, numerous additional mismatches with human sequences correspond to mouse nucleotide substitutions (Figure 1D).

In light of these findings, we systematically investigated the contribution of contaminating MEF-derived sequences to SNP calls. We mapped a similar number of reads as Avior et al. (2019) across all the studies (Figure S1D). We then applied XenofilteR, a tool previously developed for analysis of human xenografts in mice (Kluin et al., 2018). XenofilteR identifies and removes reads that map with higher efficiency to the mouse than to the human reference genome (Figure S1E). Direct comparison of samples of the same cell lines cultured with and without MEFs showed that XenofilteR detected and removed a high number of reads from co-cultures (Figure 1E). The fraction of reads removed by XenofilteR was significantly larger for naive than primed hPSC samples (Figure 1F). An independent analysis using the metagenomic tool Sequence Expression AnaLyzer (SEAL) to classify human or mouse sequences yielded similar results (Table S1). Naive cells are typically maintained at lower density than primed hPSCs, which will result in a higher contribution of MEFs in RNA-seq libraries. Variability in the representation of MEF sequences between samples likely relates to differences between cultures and laboratories in MEF preparation, relative density of hPSCs at time of harvesting, and extent to which measures are taken to deplete MEFs prior to RNA preparation. Application of XenofilteR did not significantly alter quantification of expression of the cancer-associated genes (Figure S2A). We also investigated the impact on the global transcriptome by performing principal component analysis (PCA) for all expressed protein-coding genes. This analysis (Figure S2B) showed no change in the separation of naive and primed cells on PC1, with minor shifts in distribution on PC2.

We applied the GATK for RNA-seq pipeline to all the samples, with or without application of XenofilteR (Figure S1E). We initially focused on the cancer-related SNPs identified by Avior et al. (2019). Remarkably, after depletion of mouse sequences, the number of Avior et al. (2019) SNPs fell to zero in most cases (Figure 2A; Figure S2C; Table S2). We also noticed that the number of those SNPs detectable before XenofilteR reflects the total number of mouse reads identified in each dataset (Figure 2B). A similar positive correlation (r = 0.81) was identified between the number of cancer-related SNPs identified in naive samples and the percentage of mouse reads assigned by SEAL.

Avior et al. (2019) highlighted SNPs in genes associated with signaling pathways inhibited in naive stem cell culture (*CCND2*, *HIF1a*, *FAT1*, *APC*, *BCL9L*, *MYH9*, and *CDKN1B*) and asserted that they were mutations conferring selective advantage. Every one of these SNPs was eliminated by applying XenofilteR (Table S2). Importantly, XenofilteR does not prevent detection of authentic human SNPs; >40,000 SNPs were still detected in cR-S6EOS and HNES1 samples (Figure 2C). Notably, for laminin cultures this number was not significantly changed before and after XenofilteR.

We examined the reads containing Avior et al. (2019) SNPs that were removed by XenofilteR and also those for three SNPs that remained. We aligned the reads to human and mouse reference sequences. Reads with SNPs removed by XenofilteR matched to mouse reference sequences and harbored, on average, more than four mismatches with human gene reference sequences (Figure 2D). Conversely, reads containing the three SNPs that remained after XenofilteR exhibited more mismatches with mouse than human sequences. These SNPs were in *TP53* (pR181H, pR248Q) and *CDK12* (pE131K) (Figure 2D). Both of the *TP53* SNPs were previously detected in primed hPSCs (Merkle et al., 2017). In each of the two positive datasets in this analysis, the *TP53* SNP pre-existed in the primed hPSCs and was therefore inherited by reset naive hPSCs (Table S2). The *CDK12* SNP was detected in only one of two technical replicates in a total of seven samples from Sahakyan et al. (2017) (Tables S1 and S2).

Figure 2. Elimination of Mouse Sequences Removes Cancer-Associated SNPs

(A) Number of cancer-associated SNPs from Avior et al. (2019) in different datasets, as reported in Avior et al., (2019) (red), as detected in this study without XenofilteR (blue), and as detected after removal of mouse reads using XenofilteR (grey). Error bars, SEM.

(B) Correlation between percentage of mouse reads and numbers of cancer-associated SNPs detected for all naive hPSCs in this study.

(C) Total number of SNPs before and after removal of mouse reads in cR-S6EOS and HNES1 cultures on MEF or LN.

(D) Numbers of mismatches in reads harboring the cancer-related mutation aligned against human or mouse reference sequences. Each bar represents average number of mismatches for all reads with SNPs reported by Avior et al. (2019) in naive hPSCs. n represents number of datasets with the indicated SNP. Error bars, SEM.

(E) Integrative Genome Browser screenshot of *CCND2* transcripts showing the SNP reported by Avior et al. (2019) in dashed box and nearby mismatches in reads across indicated human naive hPSC datasets.

(F) Heatmap showing number of Avior et al. (2019) SNPs detected in human naive hPSCs cultured in t2iLGö medium or PXGL medium on MEF or on LN with or without application of XenofilteR. Samples from Bredenkamp et al. (2019b) are pooled data from cultures on LN or Geltrex (GT).

The SNPs reported by Avior et al. (2019) and eliminated by XenofilteR show a very high overlap across cell lines of different genetic backgrounds cultured under different conditions and laboratories (Table S2). Incidence of identical SNPs in these circumstances would be remarkable. This is readily explained, however, by shared contamination with MEFs. For example, without the use of XenofilteR, examination of reads harboring the *CCND2* SNP revealed more than 15 single nucleotide variants that are common between different datasets and each of which matches to mouse reference (Figure 2E).

Finally, we carried out a systematic analysis of naive hPSCs cultured in our laboratory, either in our original media formulation (t2iLGö) (Takashima et al., 2014) or improved medium (PXGL) (Bredenkamp et al., 2019a; Bredenkamp et al., 2019b). In either medium, Avior et al. (2019) SNPs were detected only in cultures on MEF and all were removed by XenofilteR (Figure 2F). We then broadened the investigation to search for any other potential SNPs in tier 1 cancer genes. We uncovered only one recurrent polymorphism. A non-synonymous SNP in *ARID1A* (pI692V) was detected in HNES1 samples but was not present in any other naive cell line. *ARID1A* is frequently mutated in colon cancer, with nonsense and out-of-frame mutations (Forbes et al., 2017). However, missense mutations have not been functionally annotated. HNES1 is an embryo-derived cell line. We examined the earliest passage dataset available (Guo et al., 2016) and detected the *ARID1A* polymorphism at an allelic frequency of around 50%, as seen in the later passage samples. Notably, we did not detect this SNP in other embryo-derived cell lines, namely, HNES2 and HNES3 (Guo et al., 2016).

DISCUSSION

In summary, we find no evidence for the prevalence of cancer-related point mutations in naive hPSCs. Analysis of RNA-seq can be an effective method for identifying SNPs in hPSCs, as previously shown for certain *TP53* mutations (Merkle et al., 2017) and confirmed here. However, culture on MEF feeder layers results in the presence of mouse gene sequences in hPSC RNA-seq datasets, which can lead to erroneous SNP calls. This is particularly relevant for naive hPSCs, which in current protocols are predominantly cultured at a relatively low density on MEFs. In general, the impact of MEF sequences on gene expression is small because the majority are removed during genome alignment and because reads per gene are normalized (Figures S2A and S2B). Nonetheless, unfiltered MEF sequences can distort the measurement of genes that are lowly expressed in PSCs and highly expressed in MEF, such as *CCND2*, or can skew comparisons between hPSCs in the presence or absence of feeders. A filtration step such as XenofilteR is advisable in such cases, in particular for short read sequencing protocols with reduced quality of genome alignment.

Our analyses demonstrate that the reported detection of multiple cancer-related SNPs (Avior et al., 2019) in naive hPSCs is attributable to contamination with MEF-derived sequences. Following our report, Avior et al. have revised their methodology (Avior et al., 2020, this issue). It is essential to apply XenofilteR or an equivalent stringent quality measure to exclude mouse sequences from co-culture samples. Further analyses of naive cells under t2iLGö or PXGL culture conditions, including additional independent cultures, did not detect recurrent SNPs in any tier 1 cancer genes. Therefore, neither the generation of naive hPSCs nor their propagation imposes heightened susceptibility to point mutations in cancer-associated genes.

STAR★METHODS

Detailed methods are provided in the online version of this paper and include the following:

- KEY RESOURCES TABLE
- RESOURCE AVAILABILITY
  - Lead Contact
  - Materials Availability
  - Data and Code Availability
- EXPERIMENTAL MODEL AND SUBJECT DETAILS
  - Cell culture
- METHOD DETAILS
  - Transcriptome sequencing
- QUANTIFICATION AND STATISTICAL ANALYSIS
  - Mapping between human and mouse

SUPPLEMENTAL INFORMATION

Supplemental Information can be found online at https://doi.org/10.1016/j.stem.2020.11.014.

ACKNOWLEDGMENTS

We are grateful to James Clarke for cell culture support and to Vicki Murry and Maike Paramor for generating sequencing libraries. Sequencing was performed by the CRUK Cambridge Institute Genomics Core Facility. This research was funded by the Medical Research Council (MRC) of the United Kingdom (MR/P00072X/1). The Wellcome-MRC Cambridge Stem Cell Institute receives core support from Wellcome (203151/Z/16/Z) and MRC (MC_PC_12009). A.S. is a Medical Research Council Professor (G1100526).

AUTHOR CONTRIBUTIONS

Conceptualization, G.G.; Investigation, G.G.S.; Methodology, G.G.S.; Formal analysis, G.G.S.; Writing; G.G.S., A.S., and G.G.; Supervision, G.G. and A.S.

DECLARATION OF INTERESTS

A.S. and G.G. are inventors on a patent application relating to human naive stem cells filed by the University of Cambridge.

Received: May 21, 2020
Revised: July 23, 2020
Accepted: November 20, 2020
Published: December 14, 2020

REFERENCES

Altschul, S.F., Gish, W., Miller, W., Myers, E.W., and Lipman, D.J. (1990). Basic local alignment search tool. J. Mol. Biol. *215*, 403–410.

Anders, S., Pyl, P.T., and Huber, W. (2014). HTSeq—A Python framework to work with high-throughput sequencing data. bioRxiv. https://doi.org/10.1093/bioinformatics/btu638.

Avior, Y., Eggan, K., and Benvenisty, N. (2019). Retraction. Cell Stem Cell *28*. Published online January 7, 2021. https://doi.org/10.1016/j.stem.2020.11.020.

Avior, Y., Lezmi, E., Eggan, K., and Benvenisty, N. (2020). Cancer-Related Mutations Identified in Primed Human Pluripotent Stem Cells. Cell Stem Cell

28. Published online January 7, 2021. https://doi.org/10.1016/j.stem.2020.11.013.

Bredenkamp, N., Stirparo, G.G., Nichols, J., Smith, A., and Guo, G. (2019a). The Cell-Surface Marker Sushi Containing Domain 2 Facilitates Establishment of Human Naive Pluripotent Stem Cells. Stem Cell Reports *12*, 1212–1222.

Bredenkamp, N., Yang, J., Clarke, J., Stirparo, G.G., von Meyenn, F., Dietmann, S., Baker, D., Drummond, R., Ren, Y., Li, D., et al. (2019b). Wnt Inhibition Facilitates RNA-Mediated Reprogramming of Human Somatic Cells to Naive Pluripotency. Stem Cell Reports *13*, 1083–1098.

Dobin, A., Davis, C.A., Schlesinger, F., Drenkow, J., Zaleski, C., Jha, S., Batut, P., Chaisson, M., and Gingeras, T.R. (2013). STAR: ultrafast universal RNA-seq aligner. Bioinformatics *29*, 15–21.

Dong, C., Fischer, L.A., and Theunissen, T.W. (2019). Recent insights into the naïve state of human pluripotency and its applications. Exp. Cell Res. *385*, 111645.

Forbes, S.A., Beare, D., Boutselakis, H., Bamford, S., Bindal, N., Tate, J., Cole, C.G., Ward, S., Dawson, E., Ponting, L., et al. (2017). COSMIC: somatic cancer genetics at high-resolution. Nucleic Acids Res. *45*, D777–D783.

Guo, G., von Meyenn, F., Santos, F., Chen, Y., Reik, W., Bertone, P., Smith, A., and Nichols, J. (2016). Naive Pluripotent Stem Cells Derived Directly from Isolated Cells of the Human Inner Cell Mass. Stem Cell Reports *6*, 437–446.

Guo, G., von Meyenn, F., Rostovskaya, M., Clarke, J., Dietmann, S., Baker, D., Sahakyan, A., Myers, S., Bertone, P., Reik, W., et al. (2017). Epigenetic resetting of human pluripotency. Development *144*, 2748–2763.

Kluin, R.J.C., Kemper, K., Kuilman, T., de Ruiter, J.R., Iyer, V., Forment, J.V., Cornelissen-Steijger, P., de Rink, I., Ter Brugge, P., Song, J.-Y., et al. (2018). XenofilteR: computational deconvolution of mouse and human reads in tumor xenograft sequence data. BMC Bioinformatics *19*, 366.

Lê, S., Josse, J., and Husson, F. (2008). FactoMineR: An R package for multivariate analysis. J. Stat. Softw. *25*, 1–18.

Li, H., Handsaker, B., Wysoker, A., Fennell, T., Ruan, J., Homer, N., Marth, G., Abecasis, G., and Durbin, R. (2009). The Sequence Alignment/Map format and SAMtools. Bioinformatics *25*, 2078–2079.

McKenna, A., Hanna, M., Banks, E., Sivachenko, A., Cibulskis, K., Kernytsky, A., Garimella, K., Altshuler, D., Gabriel, S., Daly, M., and DePristo, M.A. (2010). The Genome Analysis Toolkit: a MapReduce framework for analyzing next-generation DNA sequencing data. Genome Res. *20*, 1297–1303.

Merkle, F.T., Ghosh, S., Kamitaki, N., Mitchell, J., Avior, Y., Mello, C., Kashin, S., Mekhoubad, S., Ilic, D., Charlton, M., et al. (2017). Human pluripotent stem cells recurrently acquire and expand dominant negative P53 mutations. Nature *545*, 229–233.

Robinson, J.T., Thorvaldsdóttir, H., Winckler, W., Guttman, M., Lander, E.S., Getz, G., and Mesirov, J.P. (2011). Integrative genomics viewer. Nat. Biotechnol. *29*, 24–26.

Sahakyan, A., Kim, R., Chronis, C., Sabri, S., Bonora, G., Theunissen, T.W., Kuoy, E., Langerman, J., Clark, A.T., Jaenisch, R., and Plath, K. (2017). Human Naive Pluripotent Stem Cells Model X Chromosome Dampening and X Inactivation. Cell Stem Cell *20*, 87–101.

Takashima, Y., Guo, G., Loos, R., Nichols, J., Ficz, G., Krueger, F., Oxley, D., Santos, F., Clarke, J., Mansfield, W., et al. (2014). Resetting transcription factor control circuitry toward ground-state pluripotency in human. Cell *158*, 1254–1269.

STAR★METHODS

KEY RESOURCES TABLE

REAGENT or RESOURCE	SOURCE	IDENTIFIER
Deposited Data		
RNA sequencing data from this study	Gene Expression Omnibus	GEO: GSE150933
Experimental Models: Cell Lines		
HNES1	Guo et al., 2016	N/A
cR-H9	Guo et al., 2017	N/A
EPC niPSC	Bredenkamp et al., 2019b	N/A
HDF16 niPSC	This study	N/A
Software and Algorithms		
STAR	Dobin et al., 2013	N/A
htseq-count	Anders et al., 2014	N/A
Samtools	(Li et al., 2009)	http://samtools.sourceforge.net/
XenofilteR	Kluin et al., 2018	https://github.com/PeeperLab/XenofilteR
R		https://www.R-project.org/
Genome and Genome annotation	GRCh38/mm10: Ensembl 96	http://apr2019.archive.ensembl.org/index.html
gplots		https://cran.r-project.org/web/packages/gplots/index.html
IGV	Robinson et al., 2011	http://software.broadinstitute.org/software/igv/
GATK	McKenna et al., 2010	https://gatk.broadinstitute.org/hc/en-us

RESOURCE AVAILABILITY

Lead Contact
Further information and requests for resources and reagents should be directed to and will be fulfilled by the Lead Contact, Ge Guo, g.guo@exeter.ac.uk

Materials Availability
This study did not generate new unique reagents.

Data and Code Availability
RNA-seq data from this study are deposited in Gene Expression Omnibus with accession number GEO: GSE150933.

EXPERIMENTAL MODEL AND SUBJECT DETAILS

Cell culture
Research use of hPSCs is approved by the United Kingdom Stem Cell Steering Committee.

Naive hPSCs were cultured in 5% O_2, 7% CO_2 in a humidified incubator at 37°C. Cell lines were maintained without antibiotics and confirmed free of mycoplasma contamination by periodic in-house PCR assay.

Chemically reset (cR) (Guo et al., 2017), embryo-derived (HNES) (Guo et al., 2016) and reprogrammed (niPSC) (Bredenkamp et al., 2019b) naive hPSCs were propagated in N2B27 with PXGL [1 μM PD0325901 (P), 2 μM XAV939 (X), 2 μM Gö6983 (G) and 10 ng/mL human LIF (L)] on irradiated MEF feeders as described (Bredenkamp et al., 2019a). ROCK inhibitor (Y-27632) and Geltrex (0.5μL per cm^2 surface area; hESC-Qualified, Thermo Fisher Scientific, A1413302,) were added to media during replating. Cultures were passaged by dissociation with Accutase (Biolegend, 423201) every 3-5 days.

METHOD DETAILS

Transcriptome sequencing
Total RNA was extracted from two biological replicate cultures of each cell line and time point using TRIzol/chloroform (Thermo Fisher Scientific, 15596018), and RNA integrity assessed by Qubit measurement and RNA nanochip Bioanalyzer. Ribosomal RNA was depleted from 1 μg of total RNA using Ribozero (Illumina kit). Sequencing libraries were prepared using the TruSeq RNA Sample

Prep Kit (RS-122-2001, Illumina). Sequencing was performed on the Novaseq S1 or S2 platform (Illumina) by the CRUK Cambridge Institute Genomics Core Facility.

QUANTIFICATION AND STATISTICAL ANALYSIS

Alignment was performed using the Genome build hg38 for human and Genome build mm10 for mouse. STAR (Dobin et al., 2013) was used for aligning reads. Ensembl release 96 was used to guide gene annotation in both species. Trim Galore! (http://www.bioinformatics.babraham.ac.uk/projects/trim_galore/) was used to remove adaptor contamination, if present. Best practice for variant calling in RNA-seq pipeline was used (https://gatk.broadinstitute.org/hc/en-us) (FIG.S1A, FIG.S1E), together with dbSNP146 downloaded from GATK resource bundle repository (https://gatk.broadinstitute.org/hc/en-us/articles/360035890811-Resource-bundle).

R package XenofilteR (Kluin et al., 2018) compared alignment quality between human and mouse mapped reads and filtered out sequences with higher mapping efficiency in mouse.

We quantified alignments to gene loci with htseq-count (Anders et al., 2014) based on annotation from Ensembl 96. PCA were computed on FPKM/RPKM $\log_2$ normalized counts using all the expressed protein coding genes and R library FactoMineR (Lê et al., 2008). Integrative Genomics Viewer (IGV) was used to visualize aligned reads and coverage.

Cancer-related genes and SNP location was downloaded from Table S2 in Avior et al. (2019).

Damaging and non-synonymous SNPs in coding regions were annotated using SNPnexus (SNPnexus: assessing the functional relevance of genetic variation to facilitate the promise of precision medicine) and COSMIC database (https://cancer.sanger.ac.uk/cosmic)

Mapping between human and mouse

Reads harboring the mutations were retrieved with samtools (http://www.htslib.org/doc/samtools.html). The reads were subsequently aligned using Clustal Omega webtool (https://www.ebi.ac.uk/Tools/msa/clustalo/) against the human and mouse reference. Human reference was obtained by selecting the 50 bp before and after the mutations. This 100 bp fragment was then aligned to mouse using blastn (Altschul et al., 1990) in order to identify the syntenic mouse reference region.

During alignment of reads harboring the mutations to human and mouse reference, only aligned fragments longer than 45 bp were retained to compute number of mismatches and percentage of mapping. A seed of 8 bases was used. Sequence Expression Analyzer (SEAL) (https://jgi.doe.gov/data-and-tools/bbtools/) was used to quantify sequence abundance based on human and mouse reference genomes.

Cell Stem Cell, Volume *28*

Supplemental Information

Cancer-Related Mutations Are Not Enriched in Naive Human Pluripotent Stem Cells

Giuliano Giuseppe Stirparo, Austin Smith, and Ge Guo

SUPPLEMENTAL LEGENDS

Supplement Figure 1

(A) Schematic of the pipeline used for the identification of SNPs from RNA-seq data.Variants were intersected with 43 cancer-related SNPs from Avior (2019) before and after application of hard-filtering.

(B) Total number of cancer-associated SNPs from Avior (2019) identified in HNES1 naïve cells on MEF or laminin substrates.

(C) Overlap between cancer associated SNPs from Avior et al (2019) identified in cR-S6EOS and HNES1 cells on MEF.

(D) Scatter plots of mapped reads in Avior et al. (2019) and this study.

(E) Schematic of the pipeline used for the identification of SNPs in RNA-seq data with and without removal of mouse reads by XenofilteR.

(F) Distribution of percentage of mouse reads for all naïve and primed hPSC samples.

Supplement Figure 2

(A) Heatmap with log2 expression value for cancer-associated genes in hPSCs before (CTRL) and after removal of mouse reads (XEN).

(B) PCA plots computed for all samples with all expressed protein coding genes. Left panel, before removal of mouse reads, right panel, after XenofilteR. N, naïve; P, primed as assigned by Avior et al. (2019). Purported naïve samples from Chan, Lee and Sperber align with conventional primed cells, as noted in previous analyses (Bredenkamp et al., 2019b; Takashima et al., 2014)

(C) Total number of cancer-associated SNPs detected in naïve hPSCs with different analyses.

Supplement Table 1

Summary of SNP analysis, showing datasets and samples analysed in this study and in Avior (2019). na denotes not analysed by Avior (2019). Note: cancer-related SNPs denote the 43 SNPs reported in Avior (2019)

Supplement Table 2

Table showing sample distribution of the 43 cancer-associated SNPs identified in Avior (2019) as determined in this study. The list of SNPs was downloaded from Supplementary Table 2 in Avior (2019) and include SNPs identified in hESC, iPSC or mesenchymal stromal cell (MSC) samples.

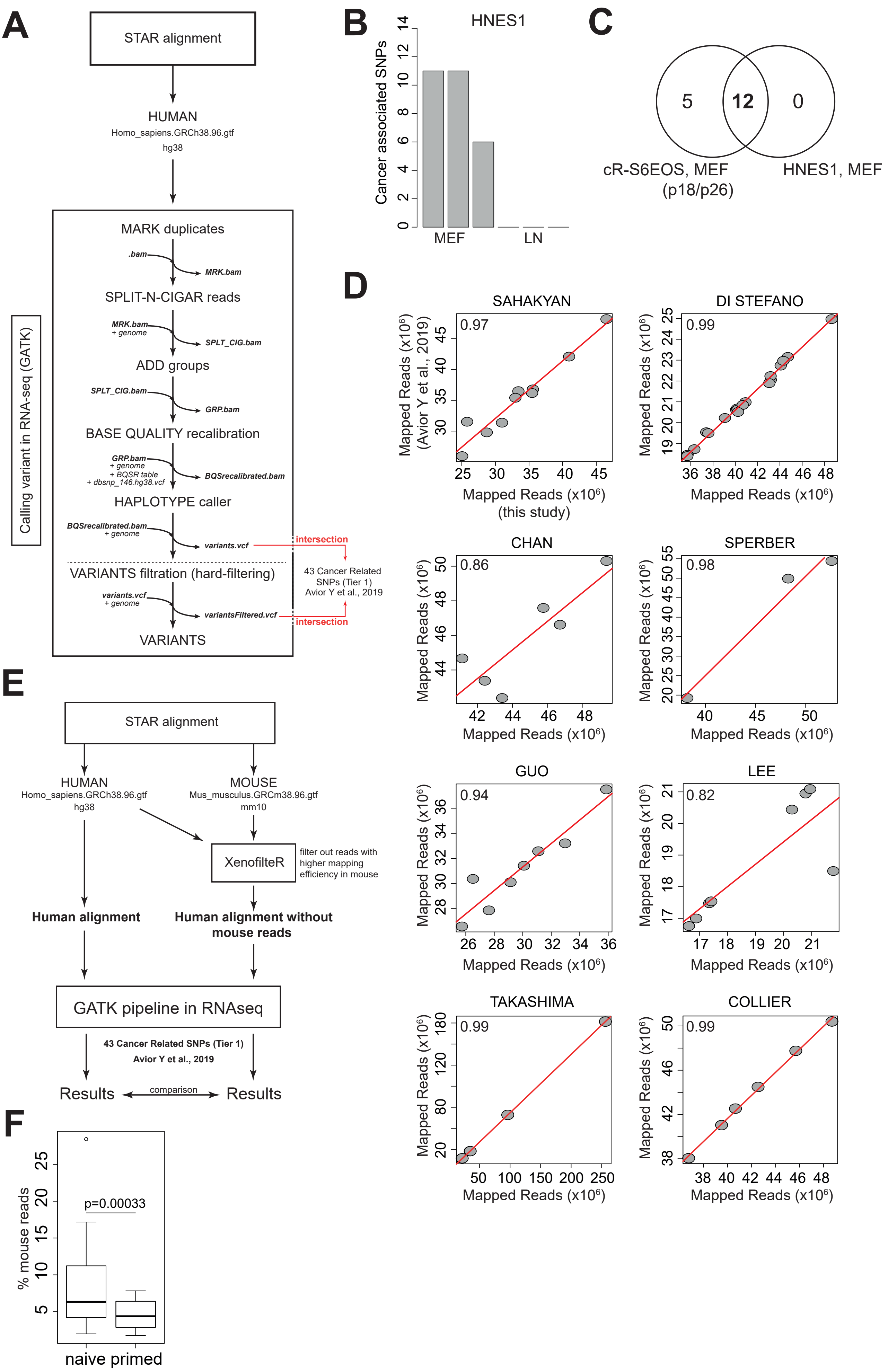

A
STAR alignment
HUMAN
Homo_sapiens.GRCh38.96.gtf
hg38
Calling variant in RNA-seq (GATK)
MARK duplicates
.bam
MRK.bam
SPLIT-N-CIGAR reads
MRK.bam + genome
SPLT_CIG.bam
ADD groups
SPLT_CIG.bam
GRP.bam
BASE QUALITY recalibration
GRP.bam + genome + BQSR table + dbsnp_146.hg38.vcf
BQSrecalibrated.bam
HAPLOTYPE caller
BQSrecalibrated.bam + genome
variants.vcf
intersection
43 Cancer Related SNPs (Tier 1) Avior Y et al., 2019
VARIANTS filtration (hard-filtering)
variants.vcf + genome
variantsFiltered.vcf
intersection
VARIANTS
B
HNES1
Cancer associated SNPs
MEF
LN
C
5
12
0
cR-S6EOS, MEF (p18/p26)
HNES1, MEF
D
SAHAKYAN
0.97
Mapped Reads (x10⁶) (Avior Y et al., 2019)
Mapped Reads (x10⁶) (this study)
DI STEFANO
0.99
CHAN
0.86
SPERBER
0.98
GUO
0.94
LEE
0.82
TAKASHIMA
0.99
COLLIER
0.99
Mapped Reads (x10⁶)
E
STAR alignment
HUMAN
Homo_sapiens.GRCh38.96.gtf
hg38
MOUSE
Mus_musculus.GRCm38.96.gtf
mm10
XenofilteR
filter out reads with higher mapping efficiency in mouse
Human alignment
Human alignment without mouse reads
GATK pipeline in RNAseq
43 Cancer Related SNPs (Tier 1)
Avior Y et al., 2019
Results
comparison
Results
F
% mouse reads
p=0.00033
naive primed

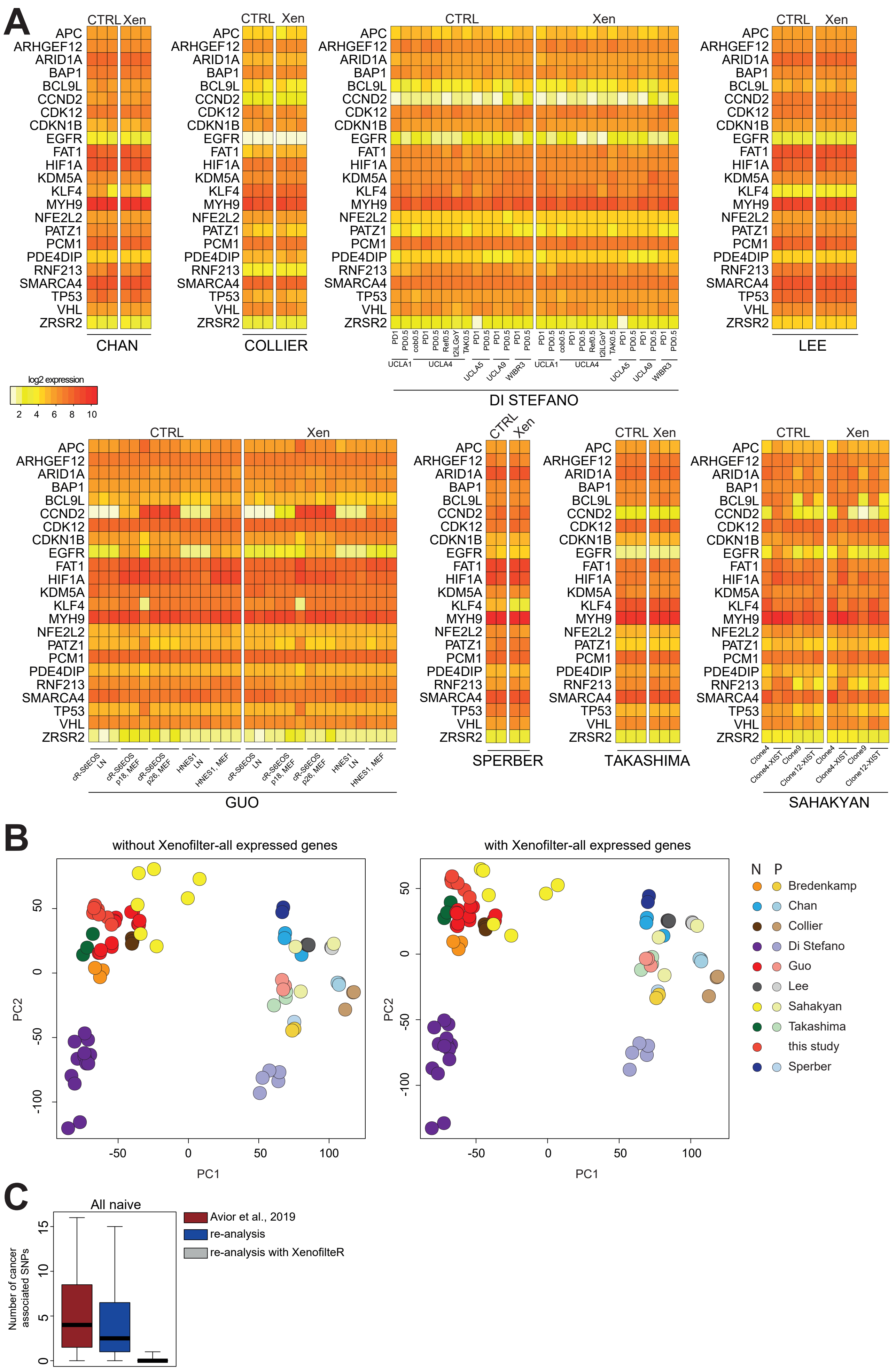
A
CTRL
Xen
APC
ARHGEF12
ARID1A
BAP1
BCL9L
CCND2
CDK12
CDKN1B
EGFR
FAT1
HIF1A
KDM5A
KLF4
MYH9
NFE2L2
PATZ1
PCM1
PDE4DIP
RNF213
SMARCA4
TP53
VHL
ZRSR2
CHAN
COLLIER
DI STEFANO
LEE
GUO
SPERBER
TAKASHIMA
SAHAKYAN
log2 expression
2 4 6 8 10
B
without Xenofilter-all expressed genes
with Xenofilter-all expressed genes
PC1
PC2
N P
Bredenkamp
Chan
Collier
Di Stefano
Guo
Lee
Sahakyan
Takashima
this study
Sperber
C
All naive
Number of cancer associated SNPs
Avior et al., 2019
re-analysis
re-analysis with XenofilteR
