# Supplementary material for: Cancer-Related Mutations Are Not Enriched in Naive Human Pluripotent Stem Cells
Source: Cell Stem Cell. 2021 Jan 7;28(1):164–169.e2. doi: 10.1016/j.stem.2020.11.014 (PMC7837212; doi:10.1016/j.stem.2020.11.014)
Supplement: Table S2. Table Showing Sample Distribution of the 43 Cancer-Associated SNPs Identified in Avior et al. (2019) as Determined in This Study — The list of SNPs was downloaded from Supplementary Table 2 in Avior et al. (2019) and include SNPs identified in hESC, iPSC or mesenchymal stromal cell (MSC) samples. [file mmc3.pdf]

													Without XenofilteR																With XenofilteR															
													Naïve								Primed								Naïve								Primed							
Gene Name	Chromosome	Position	Reference	Alternative	Amino Acid change	Number of SNPs in Avior et al., 2019	Cell type in Avior et al., 2019	Total number SNPs NAÏVE condition	Total number SNPs PRIMED condition	Total number SNPs NAÏVE condition (XenofilteR)	Total number SNPs PRIMED condition (XenofilteR)	Number of NAÏVE datasets with mutations	Chan	Collier	Di Stefano	Guo	Sahakyan	Sperber	Takashima	Lee	Chan	Collier	Di Stefano	Guo	Sahakyan	Sperber	Takashima	Lee	Chan	Collier	Di Stefano	Guo	Sahakyan	Sperber	Takashima	Lee	Chan	Collier	Di Stefano	Guo	Sahakyan	Sperber	Takashima	Lee
APC	5	112839529	G	T	p.G1312V	2	hESC	1	0	0	0	1	0	0	0	0	0	1	0	0	0	0	0	0	0	0	0	0	0	0	0	0	0	0	0	0	0	0	0	0	0	0	0	0
APC	5	112839106	G	A	p.R1171H	12	hESC/iPSC	13	0	0	0	6	1	2	0	3	2	2	3	0	0	0	0	0	0	0	0	0	0	0	0	0	0	0	0	0	0	0	0	0	0	0	0	0
ARHGEF12	11	120480206	G	A	p.R1338Q	6	hESC/iPSC	4	0	0	0	4	1	1	0	1	1	0	0	0	0	0	0	0	0	0	0	0	0	0	0	0	0	0	0	0	0	0	0	0	0	0	0	0
ARID1A	1	26697083	C	A	p.P227Q	3	hESC/iPSC	3	0	0	0	1	0	0	0	0	0	0	3	0	0	0	0	0	0	0	0	0	0	0	0	0	0	0	0	0	0	0	0	0	0	0	0	0
BAP1	3	52403788	T	G	p.K453Q	14	hESC/iPSC	3	0	0	0	2	2	0	0	0	0	1	0	0	0	0	0	0	0	0	0	0	0	0	0	0	0	0	0	0	0	0	0	0	0	0	0	0
BCL9L	11	118898575	C	G	p.S1447T	17	hESC/iPSC	10	0	0	0	5	2	0	0	1	2	2	3	0	0	0	0	0	0	0	0	0	0	0	0	0	0	0	0	0	0	0	0	0	0	0	0	0
CCND2	12	4276202	C	G	p.I131M	30	hESC/iPSC	32	3	0	0	7	2	3	11	6	7	2	1	0	0	0	0	0	3	0	0	0	0	0	0	0	0	0	0	0	0	0	0	0	0	0	0	0
CDK12	17	39462462	G	A	p.E131K	2	hESC	0	0	1	0	0	0	0	0	0	0	0	0	0	0	0	0	0	0	0	0	0	0	0	0	0	1	0	0	0	0	0	0	0	0	0	0	0
CDKN1B	12	12718204	C	A	p.P122Q	7	hESC/iPSC	0	0	0	0	0	0	0	0	0	0	0	0	0	0	0	0	0	0	0	0	0	0	0	0	0	0	0	0	0	0	0	0	0	0	0	0	0
EGFR	7	55191822	T	G	p.L858R	2	hESC	0	0	0	0	0	0	0	0	0	0	0	0	0	0	0	0	0	0	0	0	0	0	0	0	0	0	0	0	0	0	0	0	0	0	0	0	0
FAT1	4	186606105	C	G	p.V3439L	7	hESC/iPSC	12	0	0	0	3	0	0	0	7	3	2	0	0	0	0	0	0	0	0	0	0	0	0	0	0	0	0	0	0	0	0	0	0	0	0	0	0
HIF1A	14	61740857	G	C	p.A588P	29	hESC/iPSC	17	0	0	0	5	2	2	0	7	4	2	0	0	0	0	0	0	0	0	0	0	0	0	0	0	0	0	0	0	0	0	0	0	0	0	0	0
HIF1A	14	61741138	A	C	p.E681D	3	hESC/iPSC	3	0	0	0	1	0	0	3	0	0	0	0	0	0	0	0	0	0	0	0	0	0	0	0	0	0	0	0	0	0	0	0	0	0	0	0	0
KDM5A	12	354135	T	G	p.I324L	18	hESC/iPSC	12	0	0	0	5	2	0	0	5	2	1	2	0	0	0	0	0	0	0	0	0	0	0	0	0	0	0	0	0	0	0	0	0	0	0	0	0
KLF4	9	107487436	C	T	p.G320S	6	hESC/iPSC	6	0	0	0	3	3	0	1	0	0	2	0	0	0	0	0	0	0	0	0	0	0	0	0	0	0	0	0	0	0	0	0	0	0	0	0	0
MYH9	22	36349128	C	T	p.D37N	60	hESC/iPSC	9	2	0	0	5	2	0	0	1	2	1	3	0	0	0	0	0	2	0	0	0	0	0	0	0	0	0	0	0	0	0	0	0	0	0	0	0
NFE2L2	2	177234080	C	T	p.E79D	5	hESC/iPSC	3	0	0	0	3	0	1	0	0	1	1	0	0	0	0	0	0	0	0	0	0	0	0	0	0	0	0	0	0	0	0	0	0	0	0	0	0
PATZ1	22	31327054	C	G	p.G634A	2	hESC	0	0	0	0	0	0	0	0	0	0	0	0	0	0	0	0	0	0	0	0	0	0	0	0	0	0	0	0	0	0	0	0	0	0	0	0	0
PCM1	8	18025640	G	C	p.E2011Q	19	hESC/iPSC	11	1	0	0	5	1	0	0	2	3	2	3	0	0	0	0	0	1	0	0	0	0	0	0	0	0	0	0	0	0	0	0	0	0	0	0	0
PCM1	8	17939850	A	C	p.K258Q	8	hESC/iPSC	10	0	0	0	3	0	0	0	4	5	1	0	0	0	0	0	0	0	0	0	0	0	0	0	0	0	0	0	0	0	0	0	0	0	0	0	0
PDE4DIP	1	148981283	C	G	p.R901G	14	hESC/iPSC	6	3	0	0	2	1	0	0	5	0	0	0	0	0	0	0	1	2	0	0	0	0	0	0	0	0	0	0	0	0	0	0	0	0	0	0	0
RNF213	17	80345818	A	G	p.I568V	8	hESC/iPSC	11	0	0	0	4	2	0	0	5	3	1	0	0	0	0	0	0	0	0	0	0	0	0	0	0	0	0	0	0	0	0	0	0	0	0	0	0
SMARCA4	19	10987741	C	G	p.S312C	2	hESC	0	0	0	0	0	0	0	0	0	0	0	0	0	0	0	0	0	0	0	0	0	0	0	0	0	0	0	0	0	0	0	0	0	0	0	0	0
TP53	17	7675161	G	A	p.P151S	8	hESC	0	0	0	0	0	0	0	0	0	0	0	0	0	0	0	0	0	0	0	0	0	0	0	0	0	0	0	0	0	0	0	0	0	0	0	0	0
TP53	17	7675119	G	T	p.Q165K	14	hESC/iPSC	13	0	0	0	3	0	0	0	7	4	2	0	0	0	0	0	0	0	0	0	0	0	0	0	0	0	0	0	0	0	0	0	0	0	0	0	0
TP53	17	7675070	C	T	p.R181H	22	hESC	3	3	3	3	1	3	0	0	0	0	0	0	0	3	0	0	0	0	0	0	0	3	0	0	0	0	0	0	0	3	0	0	0	0	0	0	0
TP53	17	7674220	C	T	p.R248Q	12	hESC	1	4	1	4	1	0	0	0	0	0	0	0	1	0	0	0	0	0	0	0	4	0	0	0	0	0	0	0	1	0	0	0	0	0	0	0	4
TP53	17	7673821	G	A	p.R267W	14	hESC	0	0	0	0	0	0	0	0	0	0	0	0	0	0	0	0	0	0	0	0	0	0	0	0	0	0	0	0	0	0	0	0	0	0	0	0	0
TP53	17	7675148	G	C	p.T155S	13	hESC/iPSC	11	0	0	0	3	0	0	0	7	3	1	0	0	0	0	0	0	0	0	0	0	0	0	0	0	0	0	0	0	0	0	0	0	0	0	0	0
VHL	3	10142106	G	T	p.V87L	13	hESC/iPSC	4	0	0	0	3	0	0	0	1	1	0	2	0	0	0	0	0	0	0	0	0	0	0	0	0	0	0	0	0	0	0	0	0	0	0	0	0
ZRSR2	X	15822813	G	A	p.W340	16	hESC/iPSC	5	0	0	0	3	2	1	0	0	0	2	0	0	0	0	0	0	0	0	0	0	0	0	0	0	0	0	0	0	0	0	0	0	0	0	0	0
NFE2L2	2	177231324	C	T	p.E427K	5	iPSC	0	0	0	0	0	0	0	0	0	0	0	0	0	0	0	0	0	0	0	0	0	0	0	0	0	0	0	0	0	0	0	0	0	0	0	0	0
SMARCA4	19	10984319	C	G	p.I56M	12	iPSC	9	0	0	0	4	1	0	0	3	4	1	0	0	0	0	0	0	0	0	0	0	0	0	0	0	0	0	0	0	0	0	0	0	0	0	0	0
TP53	17	7670703	C	T	p.E336K	9	iPSC	10	1	0	0	3	0	0	0	4	4	2	0	0	0	0	0	1	0	0	0	0	0	0	0	0	0	0	0	0	0	0	0	0	0	0	0	0
TP53	17	7674252	C	T	p.M237I	2	iPSC	0	0	0	0	0	0	0	0	0	0	0	0	0	0	0	0	0	0	0	0	0	0	0	0	0	0	0	0	0	0	0	0	0	0	0	0	0
TP53	17	7673802	C	A	p.R273L	3	iPSC	0	0	0	0	0	0	0	0	0	0	0	0	0	0	0	0	0	0	0	0	0	0	0	0	0	0	0	0	0	0	0	0	0	0	0	0	0
TP53	17	7673776	G	A	p.R282W	3	iPSC	0	0	0	0	0	0	0	0	0	0	0	0	0	0	0	0	0	0	0	0	0	0	0	0	0	0	0	0	0	0	0	0	0	0	0	0	0
TP53	17	7674272	T	G	p.T231P	3	iPSC	0	0	0	0	0	0	0	0	0	0	0	0	0	0	0	0	0	0	0	0	0	0	0	0	0	0	0	0	0	0	0	0	0	0	0	0	0
ARID1A	1	26773393	G	A	p.G1255R	3	MSC	0	0	0	0	0	0	0	0	0	0	0	0	0	0	0	0	0	0	0	0	0	0	0	0	0	0	0	0	0	0	0	0	0	0	0	0	0
TP53	17	7675161	G	T	p.P151T	8	MSC	0	0	0	0	0	0	0	0	0	0	0	0	0	0	0	0	0	0	0	0	0	0	0	0	0	0	0	0	0	0	0	0	0	0	0	0	0
TP53	17	7673775	C	T	p.R282Q	1	MSC	0	0	0	0	0	0	0	0	0	0	0	0	0	0	0	0	0	0	0	0	0	0	0	0	0	0	0	0	0	0	0	0	0	0	0	0	0
TP53	17	7676032	A	G	p.F113I	1	MSC	0	0	0	0	0	0	0	0	0	0	0	0	0	0	0	0	0	0	0	0	0	0	0	0	0	0	0	0	0	0	0	0	0	0	0	0	0
TP53	17	7669620	C	T	p.D391N	1	MSC	0	0	0	0	0	0	0	0	0	0	0	0	0	0	0	0	0	0	0	0	0	0	0	0	0	0	0	0	0	0	0	0	0	0	0	0	0
